# Importance of developmental stage and microenvironment control in Zebrafish larvae cardiovascular studies

**DOI:** 10.1371/journal.pone.0351404

**Published:** 2026-06-12

**Authors:** Patricia Fiorino, Luigi Fernandes Rosa Cauduro, Danielle Silberspitz Konig, Leonardo Fernandes Rosa Cauduro, Caio de Araujo Santos, Juliana Alves Kavai, Isadora Durigan Duarte, Anna Laura Viacava Américo

**Affiliations:** 1 ZF HealthTech, São Paulo, Brasil; 2 Laboratório de Fisiofarmacologia Metabólica Cardiovascular e Renal, Universidade Presbiteriana Mackenzie (UPM), São Paulo, Brasil; Geosyntec Consultants Inc, UNITED STATES OF AMERICA

## Abstract

Zebrafish (*Danio rerio*) are widely used as models in cardiovascular research due to their rapid development, optical transparency, and genetic similarity to humans. However, the lack of standardized experimental conditions, particularly regarding developmental stage and microenvironmental parameters, limits reproducibility across studies. This study aimed to characterize cardiovascular function in Zebrafish larvae and evaluate the impact of developmental stage and environmental factors. Wild-type AB embryos were maintained under standard conditions, and heart rate (HR), cardiac output (CO), and ejection fraction (EF) were measured at 24, 30, 48, 52, 56, 72, 78, and 80 hours post-fertilization (hpf). The effects of variations in temperature (27.0, 27.5, and 28.0 °C) and pH (7.0, 7.4, and 8.0) were also assessed. Results showed a progressive increase in HR from 24 to 72 hpf, stabilizing thereafter. CO exhibited two phases of elevation: an early rise between 24–48 hpf and a stronger increase between 48–56 hpf. EF remained generally stable, with a transient reduction at 48 hpf. Cardiovascular performance reached a physiologically stable state after 72 hpf, defining a reliable window for functional studies. Environmental conditions modulated these parameters: temperature variation induced approximately 20% difference in HR and reduced EF, while CO was minimally affected. In contrast, pH variations within the physiological range had no significant impact on HR, CO, or EF. These findings highlight developmental and environmental variables that may influence cardiovascular measurements in Zebrafish larvae and support the development of more consistent experimental approaches in cardiovascular and toxicological research.

## Introduction

The Zebrafish (*Danio rerio*) has emerged as a powerful model organism in biomedical research, offering unique advantages for studying cardiovascular development and function [1–3]. Its small size, rapid development, optical transparency during early life stages, and genetic similarity to humans make it particularly suitable for *in vivo* imaging and functional assays [4,5].

Due to these characteristics, Zebrafish embryos and larvae have been widely used to investigate cardiac physiology, drug-induced cardiotoxicity, and the molecular mechanisms underlying cardiovascular diseases [6,7]. Furthermore, their high fecundity and easy genetic manipulation facilitate large-scale studies, making them an attractive alternative to traditional mammalian models [8,9].

Despite these advantages, a critical challenge in the field remains: the lack of standardization in experimental conditions, especially those concerning developmental stage and microenvironmental parameters [6,8,10]. The cardiovascular system of the Zebrafish undergoes rapid and dynamic morphological and functional maturation during the first seven days post-fertilization (dpf) [1,2].

The heart begins to beat around 24 hours post-fertilization (hpf), marking the onset of spontaneous contractions in a relatively simple tubular heart [11–13]. Circulation is established by 26–28 hpf [11], and by approximately 30hpf, hemodynamic forces become functionally relevant [12]. A two-chambered heart structure is fully formed by 48–72 hpf, with 48 hpf reflecting chamber organization and early atrioventricular coordination, 52–56 hpf representing progressive refinement of chamber synchronization and functional maturation, and 72 hpf corresponding to a more coordinated and functionally stable cardiac state [12,13]. Later stages such as 78–80 hpf, reflect continued functional stabilization with reduced variability in physiological parameters. However, important features such as endocardial cushion development, trabeculation, and outflow tract maturation continue progressively and remain incomplete until at least 120 hpf [12,13].

In parallel with structural development, the Zebrafish heart undergoes progressive electrical maturation, including the formation and refinement of the conduction system, action potential propagation and automaticity [14,15]. Although primitive pacemaker activity emerges as early as 24 hpf [15], full atrioventricular coordination and stabilization of electrophysiological properties such as depolarization thresholds and refractory periods develop over the subsequent days [14,15].

These electrophysiological changes influence cardiac rhythm and contractility and must be carefully considered when designing and interpreting functional cardiovascular assessments during early development [2,14]. These developmental transitions not only shape the anatomical maturation of the cardiovascular system but also result in dynamic fluctuations in physiological parameters such as heart rate, stroke volume, cardiac output and shear stress, which are frequently used as functional endpoints in experimental protocols [2,16,17].

In addition to the intrinsic fluctuations in cardiovascular function driven by developmental processes, a variety of microenvironmental factors can significantly modulate cardiac performance and vascular dynamics in Zebrafish embryos and larvae. Among these, temperature is one of the most critical variables, as Zebrafish are ectothermic organisms characterized by metabolic and physiological activities are directly influenced by ambient conditions [18,19]. Other variables such as oxygen levels, pH, osmolarity, light exposure, and anesthetics (e.g., tricaine) also modulate cardiac performance and vascular dynamics [18–20].

The literature on cardiovascular studies in Zebrafish reveals significant variation in both the developmental stages and experimental conditions, highlighting the lack of setpoints and standardized protocols. The stage of larvae at which cardiovascular parameters are assessed ranges widely, with studies often evaluating Zebrafish from 24 hpf to 5 dpf, during which the cardiovascular system is still undergoing development [16,21]. Additionally, experimental conditions such as temperature (typically ranging from 22°C to 32°C), medium composition, and immobilization techniques differ substantially across studies [16,18]. This variability in experimental design introduces challenges in comparing results and replicating experiments.

Considering the various parameters that influence cardiovascular function and the wide range of experimental protocols reported in the literature, a more detailed evaluation of cardiovascular performance at distinct developmental stages and under different microenvironmental conditions is essential [2,16]. Such evaluation would enhance the accuracy in establishing experimental setpoints, minimize variability, and improve the comparability of results across studies, thereby strengthening the application of zebrafish-based cardiovascular research in drug discovery and disease modeling [2,8]. In this context, the aim of this study is to evaluate cardiovascular function in Zebrafish larvae across early developmental time points and different microenvironmental conditions, highlighting key variables that influence experimental outcomes to support more consistent and reproducible experimental approaches.

## Materials and methods

### Zebrafish husbandry and embryo maintenance

Wild-type adult Zebrafish (*Danio rerio*, AB strain) were used for breeding to obtain fertilized eggs. Breedings between adult males and females were conducted using standard mating protocols, and embryos were collected [22]. Fertilized embryos were collected within 1 hour post-fertilization (hpf) and transferred to sterile Petri dishes with embryo medium (EM) (5.0 mM NaCl, 0.17 mM KCl, 0.33 mM CaCl₂, 0.33 mM MgSO₄), adjusted to pH 7.4. All reagents were of analytical grade and obtained from Sigma-Aldrich (St. Louis, MO, USA). Embryos were incubated in a temperature and photoperiod-controlled chamber (EL202/4, Eletrolab, Brazil) at 27 °C under a 14 h light:10 h dark cycle, according to previously described protocols [23].

Embryo viability was monitored daily throughout the experimental period. Unfertilized or non-viable embryos presenting developmental abnormalities were identified and removed from the experiment. Embryos presenting absence of heartbeat, coagulation of the embryo, lack of somite formation, or failure of tail detachment were considered non-viable and met predefined endpoint criteria. Embryos meeting endpoint criteria were euthanized immediately. No embryos died before meeting the predefined endpoint criteria.

This study was approved by the Ethics Committee on Animal Use of Universidade Presbiteriana Mackenzie (CEUA-UPM No. 03–2024) and conducted in accordance with national guidelines for the care and use of animals in research. At the conclusion of the experiments, zebrafish embryos were euthanized by immersion in an overdose of buffered tricaine methanesulfonate (MS-222; 300 mg/L). All procedures were conducted by trained personnel experienced in Zebrafish husbandry and embryo handling.

### Experimental groups

Different experimental conditions, including developmental stages and controlled microenvironmental variations, were established to evaluate the effects of intrinsic and extrinsic factors on Zebrafish embryonic cardiac activity. For each condition, embryos were obtained from multiple independent breeding events and randomly assigned to experimental groups (n = 20 embryos per group).

To assess temporal changes in cardiac activity throughout development, heartbeat measurements were performed at 24, 30, 48, 52, 56, 72, 78, and 80 hpf, corresponding to key stages of Zebrafish embryonic development [24]. At each time point, the embryo’s heartbeat was recorded, with detailed recording procedures described subsequently.

To evaluate microenvironmental variations, the effects of pH and temperature were investigated. Embryos at 24 hpf were transferred to embryo medium (EM) adjusted to pH 7.0, 7.4 and 8.0 and maintained under these conditions until 72 hpf, with daily renewal of pre-adjusted medium to ensure stable pH conditions, at which point heartbeat recordings were acquired.

Temperature effects were evaluated by maintaining embryos under baseline incubation conditions (27°C) until 72 hpf. At this developmental stage, embryos were transferred to 96-well plates and acclimated for 15 min at their respective experimental temperatures (27°C, 27.5°C, and 28°C) in a custom-designed temperature-controlled microscopy chamber with calibrated and continuously monitored temperature control, where recordings were subsequently acquired. Temperature stability was validated prior to experiments.

### Embryo’s heartbeat video acquisition

For cardiovascular assessment, viable embryos were individually transferred to 96-well plates containing EM and placed in a temperature-controlled microscopy chamber. Embryos were acclimated for 15 min at their respective experimental temperatures prior to data acquisition.

Heartbeat recordings were performed using a Nikon Eclipse TS100 inverted microscope equipped with a 20X magnification objective lens, integrated incandescent illumination, and a Basler acA2040 digital camera. Videos were recorded for 10 s at 60 frames per second, and embryos were not anesthetized or immobilized during records to avoid interference with cardiac function measurements. Videos were analyzed using DanioScope™ software to determine heart rate (HR), cardiac output (CO) and ejection fraction (EF).

### Data analysis

Videos were imported into DanioScope™ software (Noldus) for analysis. For each embryo, the region of interest (ROI) encompassing the ventricle was manually defined. The software automatically detected pixel intensity changes within the ROI over time, generating a periodic signal corresponding to cardiac cycles. Inter-beat intervals were calculated to determine HR expressed in beats per minute (bpm).

DanioScope™ also measured ventricular maximum (end-diastolic) and minimum (end-systolic) areas throughout the cardiac cycle, enabling simplified relative volume estimations from two-dimensional images. The calculation of end-systolic volume (ESV) and end-diastolic volume (EDV) was performed using the ellipsoid formula (4/3·π·a²·b), using the measurements of the longitudinal (a) and horizontal (b) axes of the ventricle (µm). Stroke volume (SV) was then obtained by the difference between ESV and EDV (EDV – ESV). The EF and CO were calculated by the following equations:


$$\begin{array}{c} \text{EF }(\%)\;=\;(\text{SV }/\text{ EDV})\;\times \text{ 100} \end{array}$$

$$\begin{array}{c} \text{CO }(\text{nL}/\text{min})\;=\text{ SV }\times \text{ HR} \end{array}$$

### Statistical analysis

The HR, EF, and CO data were organized and analyzed using GraphPad Prism version 8.3. Normality was tested with Shapiro–Wilk. Differences among developmental stages and experimental conditions (pH and temperature) were evaluated using one-way ANOVA followed by Tukey’s post hoc test for multiple comparisons. Data are presented as mean ± standard deviation. A significance level of p ≤ 0.05 was adopted.

## Results

To investigate the influence of developmental stage and microenvironmental conditions on cardiovascular physiology in Zebrafish larvae, we conducted a series of quantitative analyses focusing on heart rate progression across key developmental time points. The results presented below reflect consistent patterns observed across biological replicates and emphasize how minor variations in timing and experimental conditions can significantly affect cardiovascular parameters.

**Fig 1:**
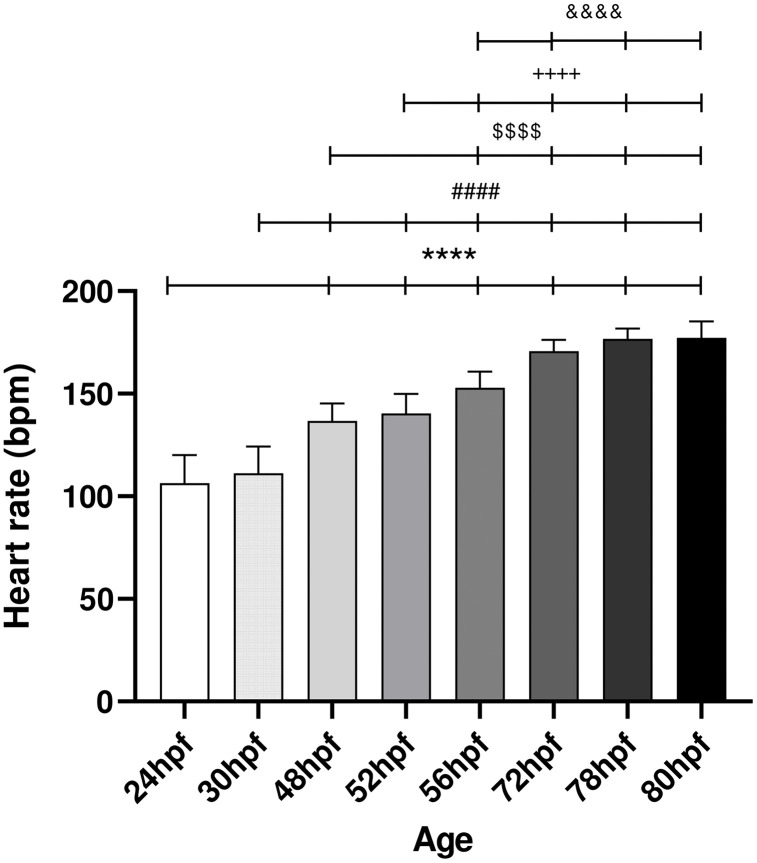
Baseline heart rate of Zebrafish embryos measured from 24 to 80 hours post-fertilization (hpf). Values are expressed in beats per minute (bpm). Data are presented as mean ± standard deviation. n = 20 per group. Significant differences were observed for p ≤ 0.05: * vs. 24hpf; # vs. 30hpf; $ vs. 48 hpf; + vs. 52 hpf; & vs. 56 hpf.

As an initial finding, a significant effect of developmental stage on heart rate was observed (F = 178.3, p < 0.0001). Heart rate increased progressively throughout early development, rising from 106 ± 14 bpm at 24 hpf to 171 ± 6 bpm at 72 hpf (p < 0.0001). No significant difference was observed between 24 and 30 hpf, whereas a marked increase occurred from 48 hpf (137 ± 8 bpm; p < 0.0001) onward compared to earlier stages. A transitional phase was observed between 48 and 52 hpf, with no significant differences between these time points, followed by a continued increase up to 72 hpf (171 ± 6 bpm; p < 0.0001). From this stage, heart rate stabilized, remaining consistent at 78 and 80 hpf (177 ± 5 bpm and 178 ± 8 bpm, respectively), with no significant differences between these later time points. These findings demonstrate a clear stage-dependent increase in cardiac activity, followed by stabilization at later developmental stages (Fig 1), highlighting the importance of accounting for developmental stage when interpreting cardiovascular function in Zebrafish larvae. Representative videos of cardiac activity across developmental stages are provided in Supporting Information (S1–S3 Videos).

**Fig 2:**
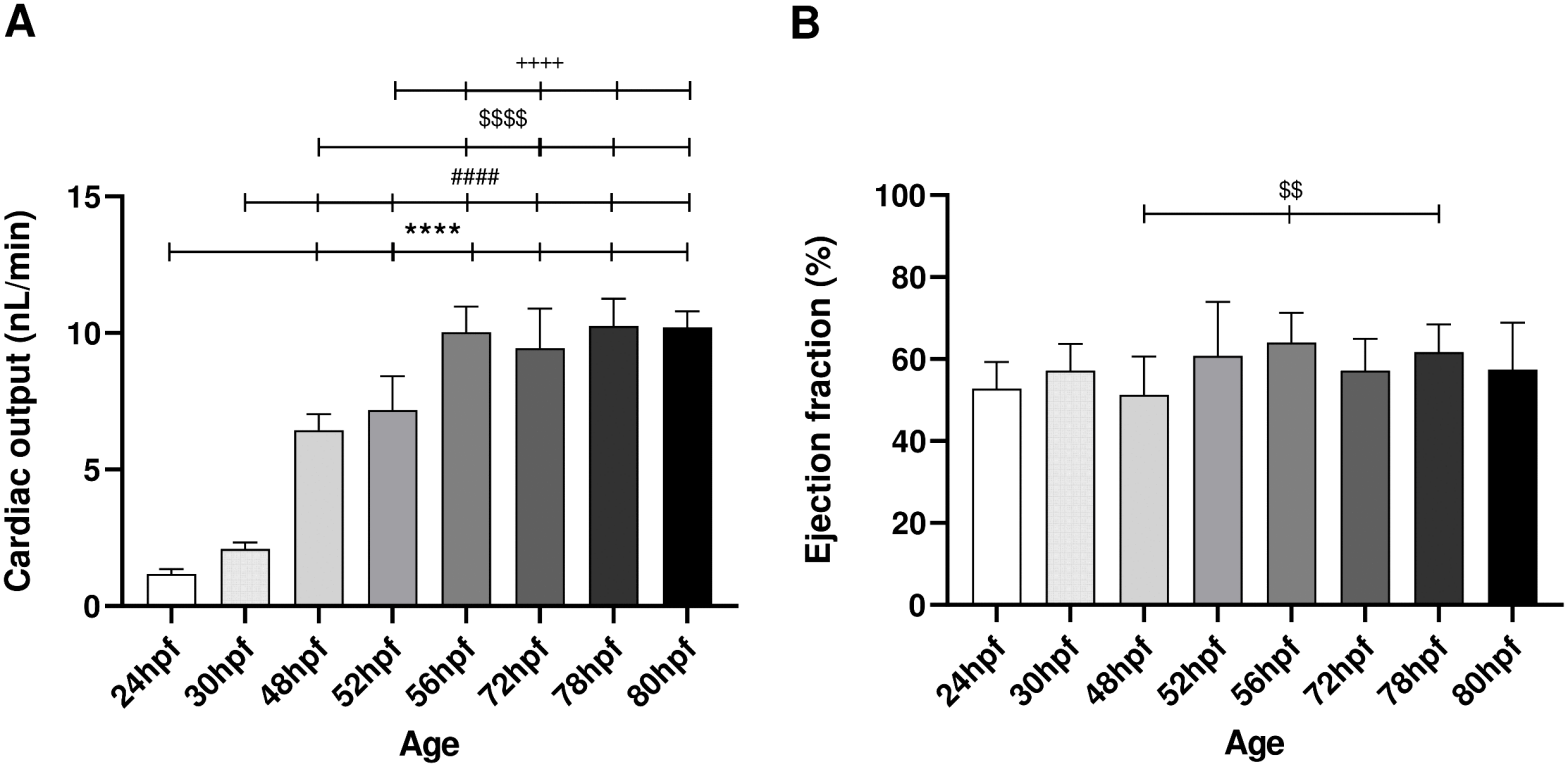
Cardiovascular function during early zebrafish development. **(A)** Cardiac output (nL/min) of zebrafish embryos measured from 24 to 80 hours post-fertilization (hpf). **(B)** Ejection fraction (%) of zebrafish embryos measured from 24 to 80 hours post-fertilization (hpf). Data are presented as mean ± standard deviation. n = 20 per group. Significant values for p ≤ 0.05: * vs. 48 hpf; # vs. 52 hpf; $ vs. 56 hpf.

To evaluate the maturation of cardiac function during early development, we quantified the cardiac output (nL/min) of Zebrafish embryos from 24 to 80 hpf (Fig 2A). A progressive increase in cardiac output was observed over time (F = 91.34, p < 0.0001), with two distinct phases of sharp elevation. The first occurred between 24 hpf (1.2 ± 0.2 nL/min) and 48 hpf (6.4 ± 0.6 nL/min; p < 0.0001), reflecting the initial establishment of functional cardiac performance. The second and most significant increase was detected between 48 and 56 hpf (10 ± 0.9 nL/min; p < 0.0001), corresponding to a critical developmental transition. From 56 hpf forward, cardiac output remained elevated and stable, with no statistically significant differences among groups 56, 72 (9.4 ± 1.5 nL/min), 78 (10.3 ± 1.0 nL/min), and 80 hpf (10.2 ± 0.6 nL/min).

Ejection fraction values remained relatively stable across most developmental time points (Fig 2B). A slight reduction was observed at 48 hpf (50.4 ± 8.9%), followed by a recovery at 52 hpf (59.7 ± 14.4%). Despite this transient variation, values at 24, 30, and from 52 hpf foward did not differ significantly, indicating a consistent level of contractile performance during both early and later stages of development.

**Fig 3:**
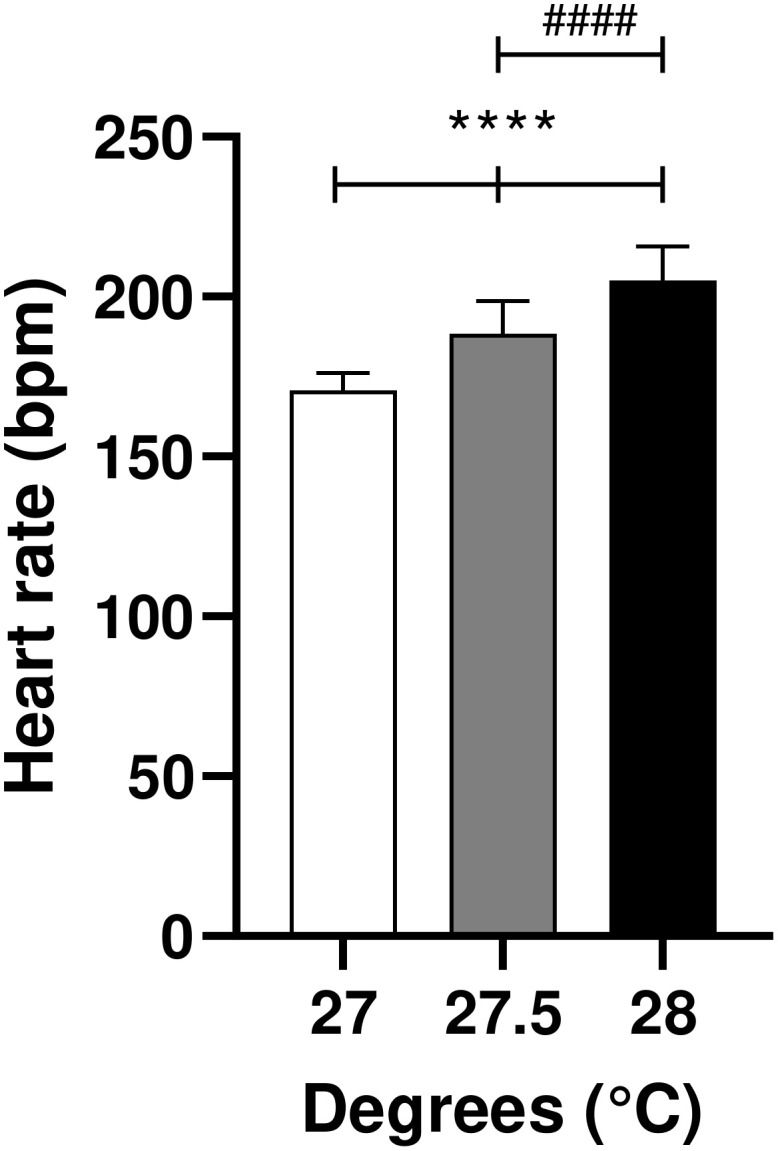
Effect of temperature on baseline heart rate in 72 hpf zebrafish embryos. Heart rate is expressed in beats per minute (bpm) and was measured at three different temperatures: 27°C, 27.5°C, and 28°C. Data are presented as mean ± standard deviation. n = 20 per group. Significant differences were observed for p ≤ 0.05: * vs. 27°C; # vs. 27.5°C.

To determine the optimal microenvironmental parameters for cardiovascular assessments, we performed an experiment in which the temperature of the embryonic medium was gradually increased in 0.5°C increments. Zebrafish embryos at 72 hpf showed a significant and progressive increase in heart rate with rising temperature (F = 69.32, p < 0.0001). Specifically, increasing the temperature from 27°C (171 ± 6 bpm) to 28°C (205 ± 11 bpm) led to a marked elevation in heart rate, reaching values approximately 20% higher than those observed at 27°C (Fig 3). These results demonstrate the strong influence of temperature on cardiac function and highlight the importance of strict thermal control during physiological evaluations in Zebrafish larvae.

**Fig 4:**
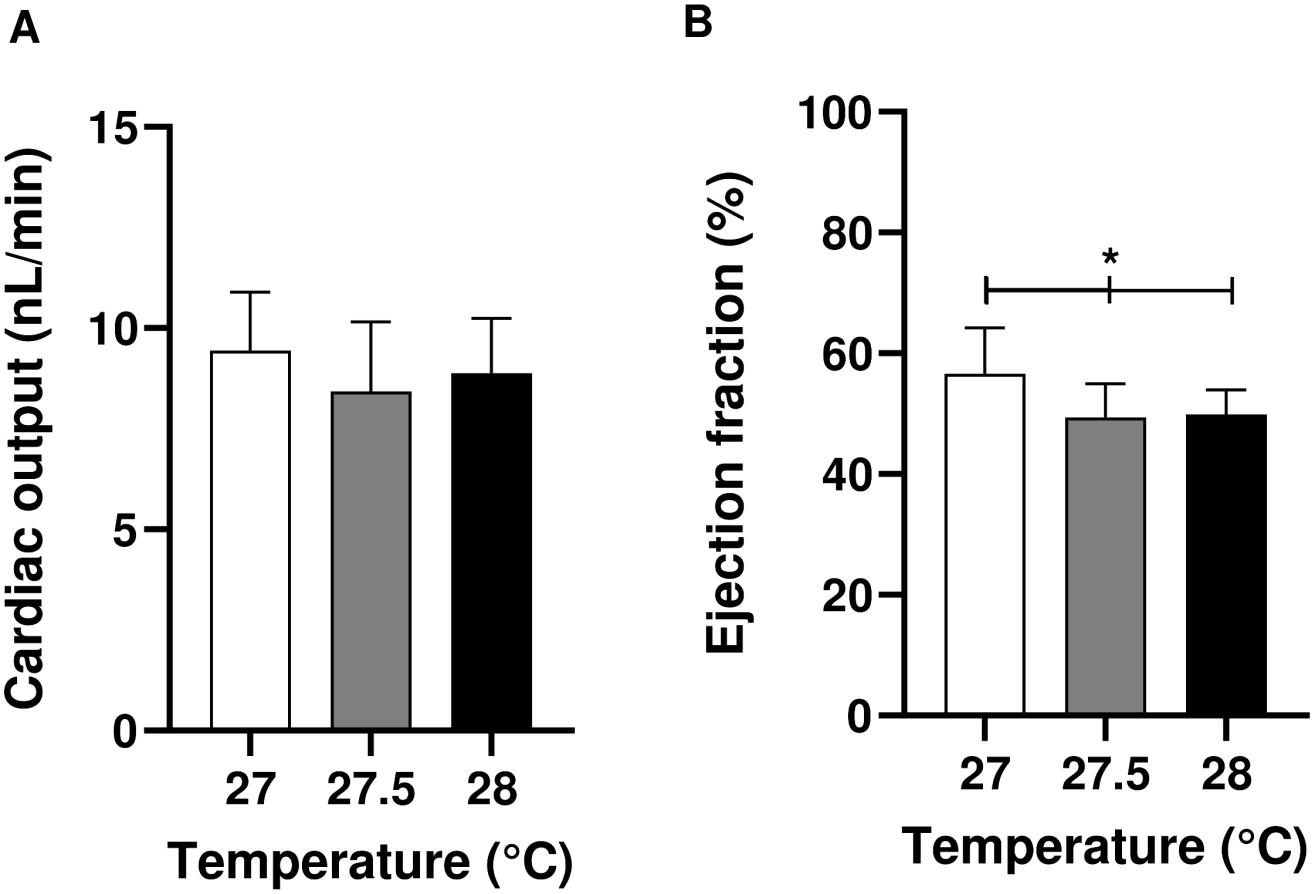
Cardiac performance parameters of Zebrafish embryos at 72 hours post-fertilization (hpf) under different incubation temperatures (27°C, 27.5°C, and 28°C). **(A)** Cardiac output (nL/min). **(B)** Ejection fraction (%). Data are presented as mean ± standard deviation. n = 20 per group. Significant values for p ≤ 0.05: * vs. 27°C.

Subsequently, we investigated the influence of incubation temperature on cardiac performance parameters in addition to heart rate (Fig 4). In Fig 4A, cardiac output (nL/min) remained relatively stable across the tested temperatures, indicating that small temperature changes did not substantially affect the volume of blood pumped per minute. In contrast, Fig 4B shows that the ejection fraction (%) was significantly reduced at 27.5°C (49.4 ± 5.6%) and 28°C (49.9 ± 4%) compared to 27°C (56.6 ± 7.5%). These results suggest that even mild fluctuations in temperature can modulate cardiac efficiency, particularly impacting systolic function in Zebrafish embryos.

Finally, we assessed whether different pH levels of the embryonic medium could influence cardiac physiology in Zebrafish embryos. Considering the relevance of environmental pH for embryonic development and its potential effects on cardiovascular function, we conducted an experiment exposing 72 hpf embryos to three pH conditions within the acceptable range recommended by regulatory agencies: pH 7.0, 7.4, and 8.0.

**Fig 5:**
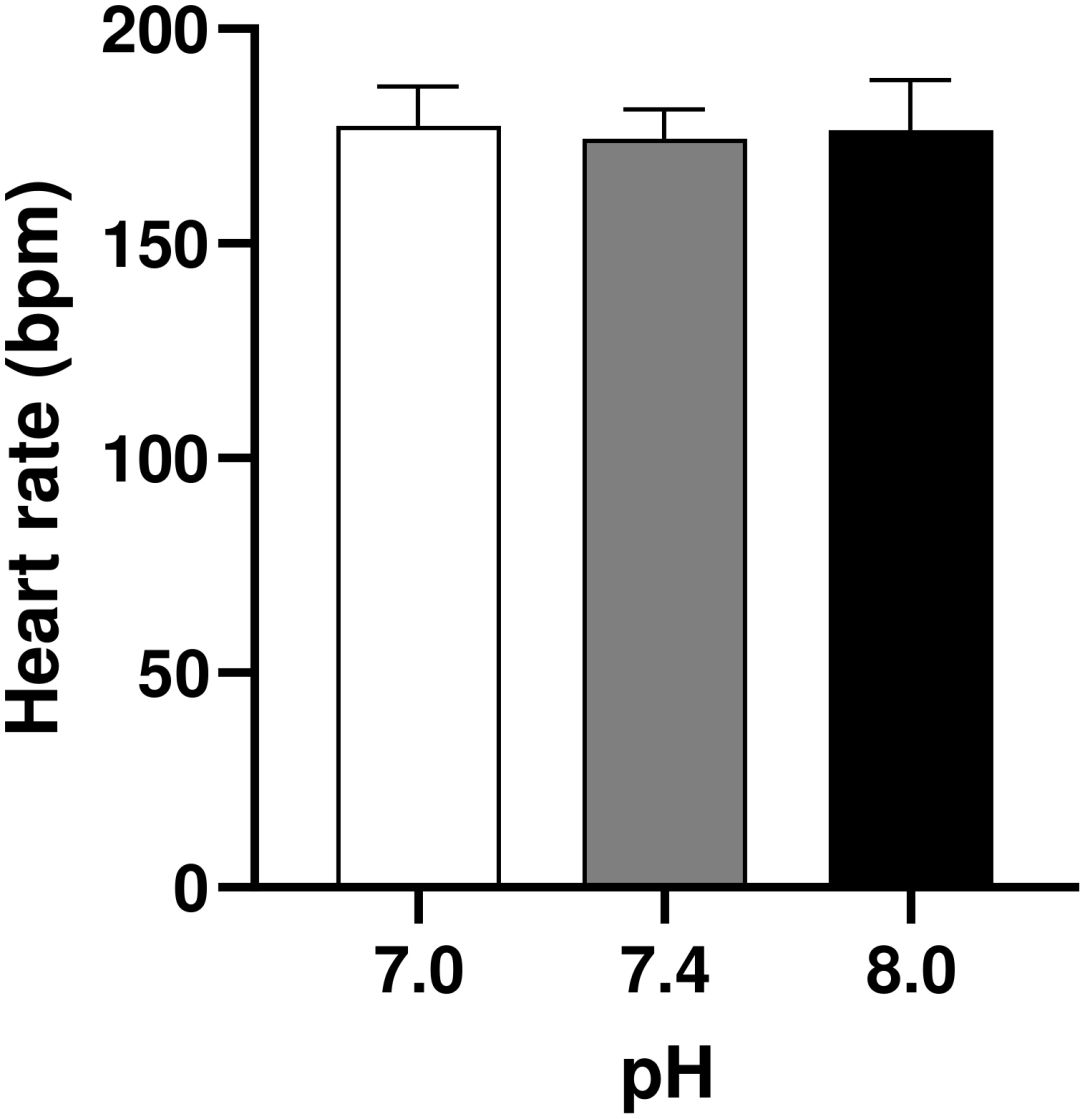
Effect of pH on baseline heart rate in 72 hpf zebrafish embryos. Heart rate is expressed in beats per minute (bpm) and was measured at three different pH levels: 7.0, 7.4, and 8.0. Data are presented as mean ± standard deviation. n = 20 per group.

**Fig 6:**
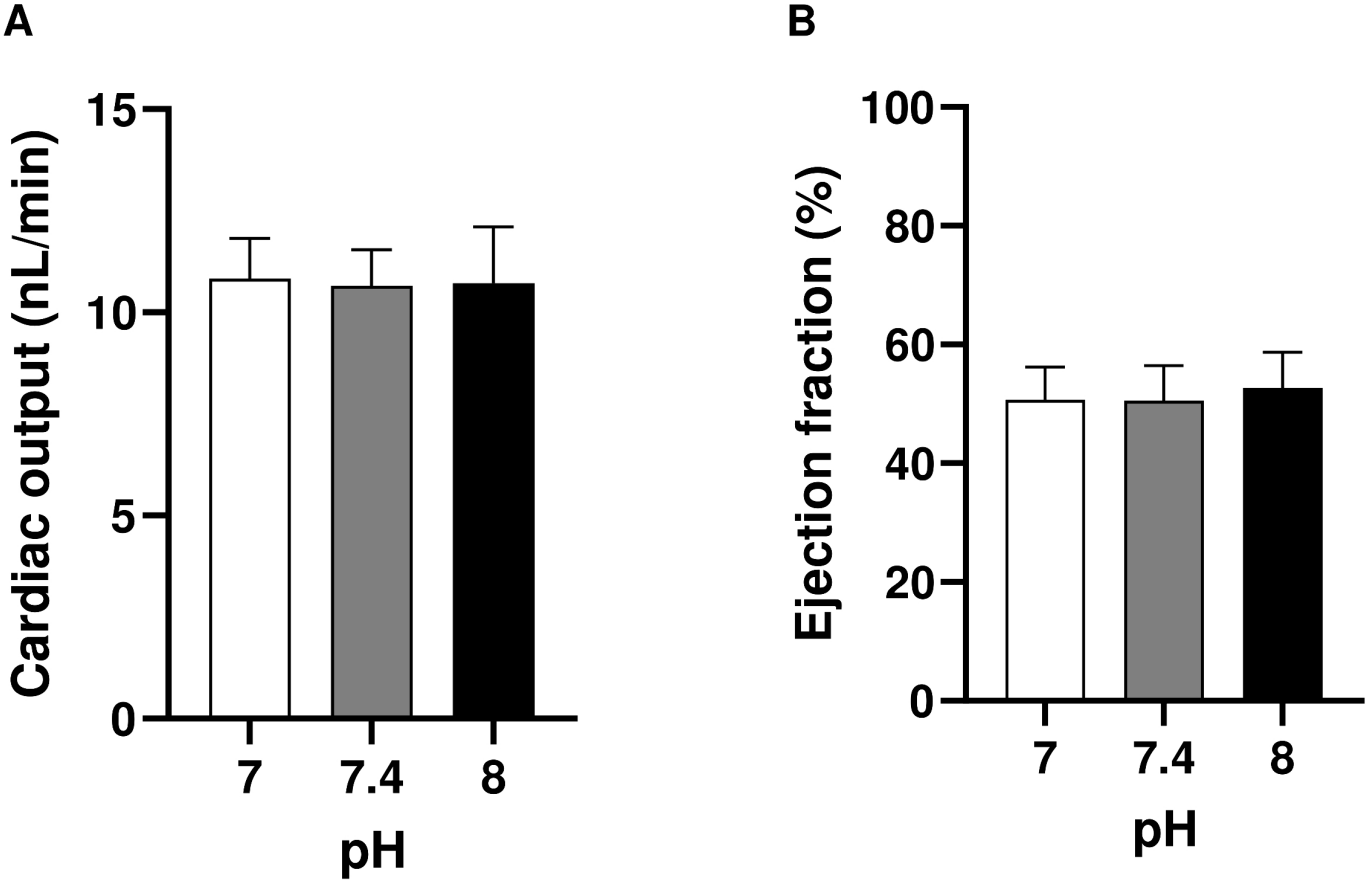
Cardiac performance parameters of zebrafish embryos at 72 hours post-fertilization (hpf) under different at three different pH levels: 7.0, 7.4, and 8.0. **(A)** Cardiac output (nL/min). **(B)** Ejection fraction (%). Data are presented as mean ± standard deviation. n = 20 per group.

Our findings showed no significant differences in heart rate among the groups (Fig 5). Heart rates remained stable across all tested conditions, indicating that pH variations within this physiological range do not significantly impact cardiac function in Zebrafish embryos at this developmental stage. Similarly, no significant differences were observed in cardiac output or ejection fraction (Fig 6A and 6B, respectively).

## Discussion

The findings presented in this study highlight the critical importance of both developmental stage and microenvironmental conditions in cardiovascular physiology in Zebrafish embryos. Our results demonstrate that even small variations in developmental timing or environmental parameters can significantly influence cardiac function, with direct implications for experimental design and reproducibility [1,2,16].

The selection of developmental time points (24–80 hpf) was based on their correspondence to key transitions in cardiac development, including the onset of spontaneous contractions (24 hpf), establishment of circulation (26–30 hpf), chamber formation and early atrioventricular coordination (48 hpf), progressive functional maturation (52–56 hpf), and the attainment of a more stable physiological state (≥72 hpf) [11–13,24]. This approach aimed to capture critical windows of functional change rather than provide a purely chronological description of development.

Our results reveal a developmental trajectory in Zebrafish cardiac function characterized by a progressive increase in heart rate between 24 and 72 hpf, followed by a plateau extending to 80 hpf. This developmental pattern is consistent with the sequential maturation of cardiac structure and electrophysiology that takes place during early Zebrafish development [12,13,24]. From 24 hpf forward, the primitive linear heart tube initiates spontaneous contractions and undergoes rightward looping, forming morphologically distinct atrial and ventricular chambers by approximately 48 hpf [12,13]. During this time, endocardial cushions begin to develop, setting the foundation for future valvular structures that will regulate intracardiac flow [25,26]. Between 48 and 72 hpf, further remodeling leads to the formation of valve-like structures in the atrioventricular (AV) canal and trabeculation of the ventricular myocardium, improving both filling dynamics and contractile output [14,27].

Importantly, the period between 48 and 72 hpf also encompasses the hatching window, during which embryos transition from the chorion-confined environment to free-swimming larvae. This transition is associated with changes in mechanical constraints, oxygen availability, and metabolic demand, which may contribute to the marked shifts in cardiac output and functional parameters observed in this interval [16,21].

Electrophysiologically, the period from 24 to 72 hpf is marked by the establishment and refinement of the cardiac conduction system. Early spontaneous contractions at 24 hpf are irregular and myogenic, but by 36–48 hpf, the sinoatrial (SA) pacemaker region becomes functionally dominant, initiating rhythmic atrial depolarization [15,28]. The AV ring starts to delay conduction between atrium and ventricle around 48 hpf, enabling sequential chamber contraction and effective circulation [14]. Between 48 and 72 hpf, the increasing expression and organization of ion channels (e.g., scn5a, cacna1c) and connexins (e.g., cx43) further enhance conduction velocity and electromechanical coupling [28,29], contributing to the observed rise in heart rate during this developmental window.

In addition to the heart rate data, we identified two distinct phases of increased cardiac output (CO) during development. The first occurred between 24 and 48 hpf, coinciding with the transition from primitive peristaltic contractions to the emergence of morphologically distinct cardiac chambers and a more coordinated atrioventricular contraction. Although myocardial contractility at this stage remains immature, the increase in CO likely reflects a combination of rising heart rate and incremental improvements in stroke volume due to chamber expansion and enhanced hemodynamic flow [30].

The second, more pronounced phase of increased CO was observed between 48 and 56 hpf, a period associated with structural and functional maturation of the heart. This includes the initial formation of valve primordia, refinement of the conduction system, and progressive synchronization between atrial and ventricular contraction. The significant rise in CO during this window likely results from both an increase in stroke volume and a transient improvement in systolic function as the embryonic myocardium begins to operate more efficiently [31].

Interestingly, ejection fraction (EF) exhibited a distinct pattern during this interval. A transient decrease was observed at 48 hpf, followed by recovery at 52 hpf, indicating a momentary reduction in systolic efficiency. This reduction likely reflects the physiological challenges imposed by the ongoing remodeling process, such as chamber differentiation, dynamic changes in ventricular compliance, and incomplete valvular function, which temporarily disrupt optimal ejection dynamics [30]. Once these structures become more organized and functionally integrated, as observed by 52 hpf, EF levels return.

Taken together, the trajectories of cardiac output and ejection fraction reflect the coordinated structural, mechanical, and electrophysiological maturation of the embryonic heart. The stabilization of heart rate, CO, and EF between 56 and 72 hpf marks the establishment of a physiologically mature cardiac phenotype, capable of sustaining the metabolic demands of larval development with consistent performance. Given this critical maturation period, functional cardiovascular assessments are most appropriately performed after 72 hpf to reduce developmental variability and enhance the reproducibility of experimental outcomes. Considering the developmental context in experimental design strengthens data reliability and reinforces the Zebrafish embryo as a robust and translationally relevant model for preclinical cardiovascular research.

One of the most striking aspects of our study was the pronounced sensitivity of cardiovascular function to minor fluctuations in environmental temperature, underscoring the critical importance of strict thermal control during experimental procedures. Even a modest increase from 27°C to 28°C at 72 hpf resulted in approximately a 20% elevation in heart rate, demonstrating the strong thermosensitivity of the embryonic cardiovascular system. This response is characteristic of ectothermic organisms, in which ambient temperature directly influences metabolic rate and cardiac pacemaker activity [18,19]. Despite this increase in heart rate, cardiac output remained stable, while ejection fraction significantly declined at higher temperatures. This dissociation reveals that elevated temperatures can compromise ventricular efficiency, likely due to reduced diastolic filling time and limited contractile performance of the immature myocardium.

Physiologically, the increased heart rate shortens the diastolic phase, limiting end-diastolic volume and impairing preload. In developing Zebrafish embryos, these effects are amplified by the limited compliance and contractile reserve of the embryonic heart. As a result, the heart compensates with tachycardia to maintain output, but at the cost of reduced ejection efficiency [31]. These findings reinforce that ejection fraction should not be interpreted as an isolated indicator of cardiac performance in developing Zebrafish embryos, particularly under conditions that alter heart rate and ventricular filling dynamics. Instead, cardiac function should be interpreted as the integrated interaction between heart rate, stroke volume, cardiac output, and ventricular efficiency.

Collectively, these findings emphasize that even slight variations in temperature can introduce significant variability in cardiovascular measurements. Thus, maintaining constant and well-defined thermal conditions is essential for ensuring physiological accuracy and experimental reproducibility in zebrafish-based cardiovascular studies.

The evaluation of pH influence demonstrated that values within the physiological range (pH 7.0–8.0) did not significantly alter baseline heart rate at 72 hpf. While this suggests a degree of tolerance to mild pH variation, previous studies have shown that more extreme acid-base imbalances can disrupt ion channel function, cardiac rhythm, and myocardial excitability [20]. Therefore, while our findings provide reassurance for standard laboratory conditions, they also highlight the need for future studies exploring the cardiovascular effects of pH extremes, particularly in environmental risk assessment scenarios.

## Conclusion

In conclusion, our study provides a refined framework for conducting cardiovascular assessments in Zebrafish embryo by identifying key developmental and environmental parameters that influence cardiac physiology. By identifying developmental windows of physiological stability, particularly after 72 hpf, and quantifying the effects of temperature and pH, our findings contribute to a better understanding of variables that may influence experimental variability and support the development of more consistent Zebrafish-based cardiovascular studies.

## Supporting information

S1 Video Representative cardiac activity at 24 hpf. Representative video showing early cardiac contractions in zebrafish embryos at 24 hours post fertilization maintained at 27°C. Mean heart rate: 108 bpm.
(MP4)

S2 Video Representative cardiac activity at 48 hpf. Representative video demonstrating synchronized cardiac contractions and circulation in zebrafish embryos at 48 hours post fertilization maintained at 27°C. Mean heart rate: 147 bpm.
(MP4)

S3 Video Representative cardiac activity at 72 hpf. Representative video showing stabilized cardiac function in zebrafish larvae at 72 hours post fertilization maintained at 27°C. Mean heart rate: 173 bpm.
(MP4)

S1 Table Individual and summarized cardiovascular measurements from zebrafish larvae under different developmental and microenvironmental conditions. Worksheet 1 contains the raw individual measurements collected from each larva. Worksheet 2 contains the corresponding mean ± standard deviation values used for data visualization.
(XLSX)
